# Chronically altered ventricular activation causes pro-arrhythmic cardiac electrical remodelling in the chronic AV block dog model

**DOI:** 10.1093/europace/euac164

**Published:** 2022-09-20

**Authors:** Valerie Y H van Weperen, Iris ter Horst, Albert Dunnink, Alexandre Bossu, Odette A Salden, Henriette D M Beekman, Avram Oros, Vincent Bourgonje, Thom Stams, Mathias Meine, Marc A Vos

**Affiliations:** Department of Medical Physiology, Universitair Medisch Centrum Utrecht, Yalelaan 50, 3584 CM Utrecht, The Netherlands; Department of Medical Physiology, Universitair Medisch Centrum Utrecht, Yalelaan 50, 3584 CM Utrecht, The Netherlands; Department of Medical Physiology, Universitair Medisch Centrum Utrecht, Yalelaan 50, 3584 CM Utrecht, The Netherlands; Department of Medical Physiology, Universitair Medisch Centrum Utrecht, Yalelaan 50, 3584 CM Utrecht, The Netherlands; Department of Cardiology, University Medical Center Utrecht, 3584 Utrecht, The Netherlands; Department of Medical Physiology, Universitair Medisch Centrum Utrecht, Yalelaan 50, 3584 CM Utrecht, The Netherlands; Department of Medical Physiology, Universitair Medisch Centrum Utrecht, Yalelaan 50, 3584 CM Utrecht, The Netherlands; Department of Medical Physiology, Universitair Medisch Centrum Utrecht, Yalelaan 50, 3584 CM Utrecht, The Netherlands; Department of Medical Physiology, Universitair Medisch Centrum Utrecht, Yalelaan 50, 3584 CM Utrecht, The Netherlands; Department of Cardiology, University Medical Center Utrecht, 3584 Utrecht, The Netherlands; Department of Medical Physiology, Universitair Medisch Centrum Utrecht, Yalelaan 50, 3584 CM Utrecht, The Netherlands

**Keywords:** Biventricular pacing, Ventricular arrhythmias, Sudden cardiac death, Altered ventricular activation, Chronic AV block dog model

## Abstract

**Aims:**

Altered ventricular activation (AVA) causes intraventricular mechanical dyssynchrony (MD) and impedes contraction, promoting pro-arrhythmic electrical remodelling in the chronic atrioventricular block (CAVB) dog. We aimed to study arrhythmogenic and electromechanical outcomes of different degrees of AVA.

**Methods and results:**

Following atrioventricular block, AVA was established through idioventricular rhythm (IVR; *n* = 29), right ventricular apex (RVA; *n* = 12) pacing or biventricular pacing [cardiac resynchronization therapy (CRT); *n* = 10]. After ≥3 weeks of bradycardic remodelling, Torsade de Pointes arrhythmia (TdP) inducibility, defined as ≥3 TdP/10 min, was tested with specific *I_Kr_*-blocker dofetilide (25 μg/kg/5 min). Mechanical dyssynchrony was assessed by echocardiography as time-to-peak (TTP) of left ventricular (LV) free-wall minus septum (ΔTTP). Electrical intraventricular dyssynchrony was assessed as slope of regression line correlating intraventricular LV activation time (AT) and activation recovery interval (ARI). Under sinus rhythm, contraction occurred synchronous (ΔTTP: −8.6 ± 28.9 ms), and latest activated regions seemingly had slightly longer repolarization (AT–ARI slope: −0.4). Acute AV block increased MD in all groups, but following ≥3 weeks of remodelling IVR animals became significantly more TdP inducible (19/29 IVR vs. 5/12 RVA and 2/10 CRT, both *P* < 0.05 vs. IVR). After chronic AVA, intraventricular MD was lowest in CRT animals (ΔTTP: −8.5 ± 31.2 vs. 55.80 ± 20.0 and 82.7 ± 106.2 ms in CRT, IVR, and RVA, respectively, *P* < 0.05 RVA vs. CRT). Although dofetilide steepened negative AT–ARI slope in all groups, this heterogeneity in dofetilide-induced ARI prolongation seemed least pronounced in CRT animals (slope to −0.8, −3.2 and −4.5 in CRT, IVR and RVA, respectively).

**Conclusion:**

Severity of intraventricular MD affects the extent of electrical remodelling and pro-arrhythmic outcome in the CAVB dog model.

## Graphical Abstract

**Graphical Abstract:**
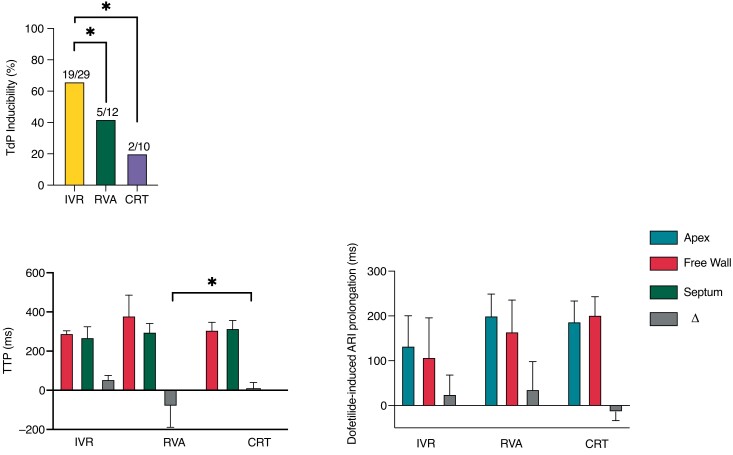
After complete atrioventricular block, chronic atrioventricular block (CAVB) dogs were exposed to different extents of altered ventricular remodelling. Animals either remodelled under idioventricular rhythm (IVR), right ventricular apex (RVA) pacing or cardiac resynchronization therapy (CRT). After remodelling, mechanical dyssynchrony was assessed using echocardiography as difference in time-to-peak (TTP) of left ventricular (LV) free-wall and LV septum. Dofetilide was also administered to assess Torsade de Pointes arrhythmia (TdP) inducibility and dofetilide-induced activation recovery interval (ARI) prolongation. Severity of intraventricular mechanical dyssychrony was shown to affect the extent of electrical remodelling and pro-arrhythmic outcome in the CAVB dog model.

**What’s new?**

- Altered ventricular activation (AVA) causes electromechanical dyssynchrony, which impedes contraction and thereby induces ventricular electrical remodelling. Even though these remodelling processes improve cardiac output, they also cause an increased susceptibility for ventricular arrhythmias.
- In the chronic atrioventricular block (CAVB) dog model, increased mechanical dyssynchrony correlates with increased left ventricular electrical dyssynchrony (reflected by intraventricular dispersion of repolarization).
- Increased intraventricular dispersion of repolarization was associated with higher inducibility rates for Torsade de Pointes arrhythmias.
- Therefore, AVA contributes to pro-arrhythmic remodelling in the CAVB dog model, as the degree of electromechanical dyssynchrony correlates with the arrhythmogenic outcome.

## Introduction

A prolonged QRS complex is associated with higher all-cause mortality and possibly fatal, ventricular arrhythmias in heart failure (HF) patients.^1^ Therefore, an increasing number of HF patients are receiving cardiac resynchronization therapy (CRT), also known as biventricular pacing. Cardiac resynchronization therapy aims to restore ventricular electromechanical synchrony and can reduce morbidity and mortality in HF patients.^2^ Moreover, the BLOCK-HF study showed that CRT resulted in fewer deaths and less progression of HF than in right ventricular apex (RVA) paced patients,^3^ suggesting the superiority of CRT over more conventional chronic RVA pacing.

However, although almost one-third of CRT recipients are refractory to this therapy, the pathophysiology underlying CRT ‘non-responders’ and how this outcome can be *predicted* and *prevented* remains poorly understood. Moreover, a meta-analysis by Deif *et al.*^4^ reported higher incidence of ventricular arrhythmias in non-responders compared with responders. Furthermore, Haugaa *et al.*^5^ demonstrated significant correlation between incidence of ventricular arrhythmias and CRT-associated mechanical dyssynchrony (MD). Collectively, these results suggest that pacing-induced altered ventricular activation (AVA), and consequential MD, might initiate pro-arrhythmic cardiac remodelling. Simultaneously, this would also explain the superiority of CRT over RVA,^3^ as CRT should cause less dyssynchrony than RVA and therefore induces less pro-arrhythmic remodelling. However, more research is needed into the electrical consequences of acute and chronic pacing, and the role of mechanical (dys)synchrony on promoting development of pro-arrhythmic conditions.

Studies on pacing-induced electrical remodelling can be performed in the chronic atrioventricular block (CAVB) dog, which has proven to be an outstanding paradigm to study (i) aetiological factors that predisposition to ventricular arrhythmias and sudden cardiac deaths and (ii) the electromechanical consequences of different pacing strategies.^6,7^ In this model, ablation of the AV-node and subsequent drop in cardiac output initiate several remodelling processes that cumulatively restore cardiac output.^8^ Adversely, electrical remodelling, which includes downregulation of potassium channels, results in susceptibility for Torsade de Pointes arrhythmia (TdP). Especially in combination with additional (acute) impediments of cardiac repolarization such as anaesthetics,^9^ bradycardia^10^ and/or administration of specific *I_Kr_*-blocker dofetilide,^11^ TdP inducibility is reached in ± 75% of animals.^8^

In this study, we compared the electromechanical and arrhythmogenic consequences of different extents of AVA, achieved through chronic exposure to either idioventricular rhythm (IVR), RVA or CRT conditions, in the CAVB dog model. We hypothesized that more electromechanical dyssynchrony would prompt more extensive remodelling and processes and thereby result in increased TdP susceptibility.

## Methods

All experiments were approved by ‘the Committee for Experiments on Animals’ of Utrecht University, the Netherlands. Animal handling and care was in accordance with the ‘European Directive for the Protection of Vertebrate Animals used for Experimental and Scientific Purpose European Community Directive 86/609/CEE’. In total, 51 adult purpose-bred mongrel dogs (18 males, 24 ± 3 kg, Marshall, USA) were included.

### Animal preparation

Premedication was given ± 30 min before induction of general anaesthesia and consisted of 0.5 mg/kg methadone (*i.m.*), 0.5 mg/kg acepromazine (*i.m.*), 0.02 mg/kg atropine (*i.m*.), and 0.1 mg/kg metacam (*s.c.*). Before the experiment, prophylactic antibiotic ampicillin (1000 mg) was injected (*i.v.*) and buprenorphine 0.3 mg (*i.m*) was administered after the experiments. Following the first experiment, temperature was monitored daily and amoxicillin-clavulanic acid (250 mg, *p.o.*) and metacam (0.1 mg/kg, *p.o*.) were administered for five consecutive days. General anaesthesia was induced using pentobarbital (25 mg/kg, *i.v.*) and maintained with mechanical ventilation of isoflurane (1.5%) in a mixture of O_2_ and N_2_O (1:2). In CRT-paced animals (*n* = 10), left ventricular (LV) leads of the CRT pacemaker were implanted through a right-sided thoracotomy (fourth intercostal space). All animals were implanted with leads and generators from Medtronic (Maastricht, Netherlands). In CRT-paced animals, the epicardial, LV unipolar pacing lead (5071; 53 cm) was screwed on the basal, anterolateral wall of the LV. Correct pacing and absence of adverse phrenic nerve stimulation were confirmed.

**Figure 1:**
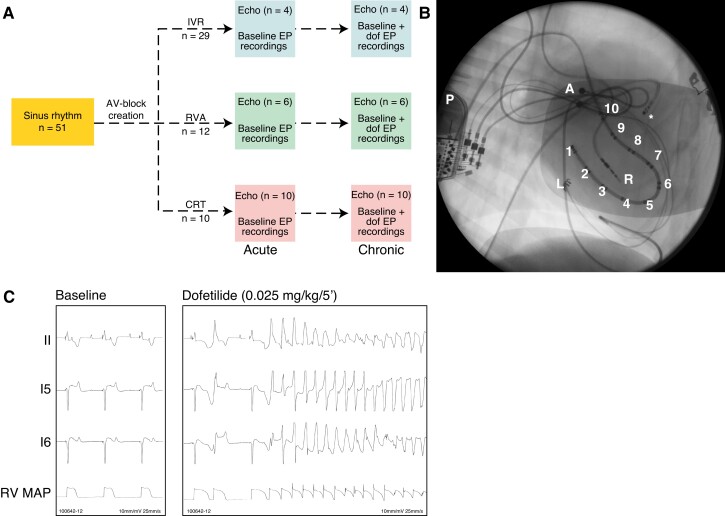
Overview of experimental methodology. (*A*) Experimental design and set-up. (*B*) Positioning of the duo-decapolar catheter in the ventricle (1–10 depict the 10 LV electrodes measuring local EGM). The asterisk (*) represents the MAP catheter in the RV MAP. The atrial lead (A), epicardial lead stimulating the LV (L), the RV pacing lead (R) and the pacemaker (P), are also depicted. Animal in left-supine position. (*C*) Representative tracings under baseline and following dofetilide infusion. Recordings from lead II, electrode 5 (I5) and 6 (I6) from the LV duo-decapolar catheter and RV MAP are shown. CRT, cardiac resynchronization therapy; IVR, idioventricular rhythm; MAP, monophasic action potential; RV, right ventricle; RVA, right ventricular apex.

Hereafter, the right ventricular (RV) bipolar pacing lead (5076; 85 cm) was introduced in CRT and RVA (*n* = 12) paced animals *via* the jugular vein and, under fluoroscopic guidance, screwed in the RVA. The RVA lead was tunnelled to the thoracic device pocket and all leads were connected to the pacemaker (different models for RVA animals–CRT-P, Consulta, C3TR01; LV-RV delay: 0 ± 8 ms for CRT animals; *Figure 1B*). Proper functioning was tested, impedance and pacing threshold determined, and the thorax closed. Next, radiofrequency ablation of the proximal His-bundle induced complete atrioventricular (AV) block in all animals.

Following the acute experiment, rate was uncontrolled in IVR (*n* = 29) animals, whereas RVA and CRT animals were paced at the lowest captured rate (55 ± 7 bpm for RVA animals; 54 ± 5 bpm for CRT animals).

### Experiments

Anaesthetized experiments including electrical and mechanical recordings were performed under sinus rhythm (SR), following acute IVR, RVA, or CRT pacing (aIVR, aRVA, or aCRT, respectively) and after remodelling under IVR, RVA, or CRT conditions had completed (cIVR, cRVA, and cCRT, respectively; *Figure 1A*).^12^ Mechanical recordings were made in four IVR, six RVA, and all CRT animals. Under experimental conditions, RVA and CRT animals were continuously paced at 60 bpm. The cIVR, cRVA, and cCRT experiments were conducted at 3.6 ± 1.0, 3.1 ± 0.4 and 2.9 ± 0.2 weeks after creation of AV block and included additional assessment of TdP inducibility in all animals. Torsade de Pointes susceptibility was determined through administration of specific *I_Kr_*-blocker dofetilide (0.025 mg/kg/5 min, *i.v.*; *Figure 1A*). Dofetilide infusion was discontinued if TdP occurred within 5 min after start of administration and persistent arrhythmias (>10 s) were manually terminated by electrical cardioversion. Electrophysiological recordings consisted of six-leads surface ECG, an endocardial RV monophasic action potential (MAP) catheter (Hugo Sachs Electronik, March-Hugstetten, Germany; not in cIVR experiments) and endocardial duo-decapolar catheter (St. Jude Medical, Veenendaal, the Netherlands), recording unipolar electrograms (EGM) from 10 distinct LV regions (*Figure 1B*). In IVR experiments, the use of an LV EGM was limited to four animals. Catheters were introduced through the femoral artery or vein and positioned under fluoroscopic guidance. Electrophysiological recordings included a 10 min baseline period and 20 min following start of dofetilide infusion.

### Echocardiography

Echocardiography was performed in a subset of the animals (*n* = 13 for SR, *n* = 4 for IVR, *n* = 6 for RVA, and *n* = 10 for CRT) and conducted under anaesthesia using a Philips iE33 ultrasound machine (Philips, Best, the Netherlands). Animals were positioned in the right-sided supine position, except for aCRT conditions. Left ventricular parasternal short-axis views were made at papillary level and appropriate beats were selected based on PQ-interval duration. Radial myocardial peak strain (PS), onset of radial strain (onset), and time-to-peak (TTP) of LV septal- and free-wall were analyzed with TomTec-Arena, 2D-Strain (TomTec, Unterschleissheim, Germany). Left ventricular septal- and free-wall regions were determined after division of the LV into six segments; regions were averages of the two adjoining segments best corresponding to the septal- and free-wall. The remaining segments, located between the septal- and free-wall sections, were discarded. The ΔPS, ΔOnset and ΔTTP were calculated by subtracting the value of the septal-wall from the free-wall (free-wall—septum).

### Electrophysiology

PP, RR, QRS, QT, and T-wave peak-to-end (Tp-e) intervals were obtained from the surface ECG and analyzed offline using EPTracer (Cardiotek, Maastricht, the Netherlands). Duration of all intervals were manually measured and averaged over five consecutive beats. QT-interval was corrected for heart rate (QTc) using the Van der Water formula.^13^ Right ventricular MAP durations (MAPDs; determined at 80% repolarization) and LV activation recovery intervals (ARIs) were determined by a custom-made Matlab application (Mathworks, Naticks, USA). Signals derived from the most distal and proximal electrode (1 and 10, respectively) of the duo-decapolar catheter were excluded from analysis because of P-wave interference. Short-term variability of repolarization (STV) was determined from 30 consecutive beats using the formula: ∑|*D_n_* + 1 – *D_n_*|/(30 × √2); D being RV MAPD or LV ARI.^14^ Interventricular dispersion of repolarization (ΔMAPD) was calculated as LV ARI—RV MAPD; the average ARI of all LV EGM was used for calculations. Activation times (ATs) of the LV EGM electrodes were manually determined as time difference between pacing spike and minimum *dV*/*dt* of the EGM. Left ventricular activation time was calculated as average AT of all LV EGM. Right ventricular activation time was measured as time difference between pacing spike and steepest upstroke of the MAP. Interventricular delay in activation (ΔAT) was calculated as LV_AT_ minus RV_AT_. Left ventricular intraventricular heterogeneity in activation and repolarization were assessed by comparing ARI and AT of the duo-decapolar catheter. Activation time and ARI of EGM electrodes were used to establish AT–ARI slopes. All electrophysiological measurements were made under baseline conditions and either before the first ectopic beat or 5 min following start of dofetilide infusion.

### Arrhythmia quantification

Torsade de Pointes were identified as polymorphic ventricular tachycardias of ≥5 beats twisting around the isoelectric line. Torsade de Pointes-inducibility was defined as ≥3 TdP within 10 min following start of dofetilide. Severity of arrhythmic activity was quantified as arrhythmia score (AS): all events were scored according to the *n* + 1 rule (*n* = number of ectopic beats in an arrhythmic event). Defibrillations were awarded 50, 75, or 100 points when single, double, or ≥ triple defibrillations were required, respectively. The score of the three most severe episodes during the 10 min following start of dofetilide were averaged to obtain a final AS.

### Statistical analysis

All results are displayed as mean ± standard deviation (SD). Repeated-measures ANOVA with *post hoc* Bonferroni correction was used to determine the statistical significances of the electrophysiological and echocardiographic data. Inducibility and AS were analyzed by McNemar’s and Wilcoxon signed rank tests, respectively. Results were considered statistically significant if *P* < 0.05.

## Results

### Sinus rhythm and acute atrioventricular block

**Figure 2:**
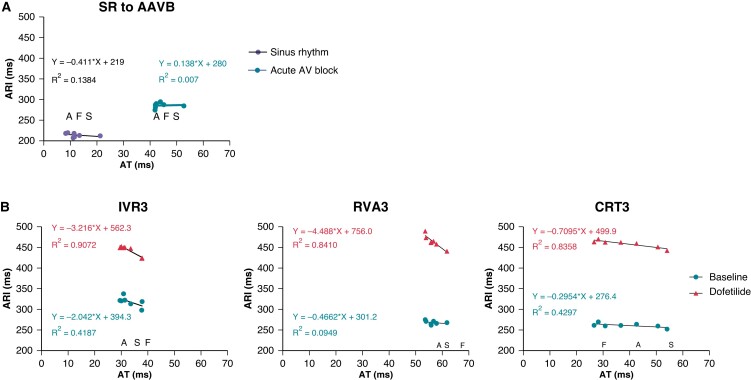
LV endocardial duo-decapolar catheter and the dynamical relationship between LV activation and repolarization over time. The relationship between local LV ATs and corresponding ARIs in (*A*) SR and following acute AV block and (*B*) following 3 weeks of remodelling under the respective rate strategies. Order of LV activation are represented by the letters A (Apex), F (Free-wall), and S (Septum). Baseline measurements are blue, and dofetilide measurements are red. *N* = 14 for SR, *n* = 26 for AAVB, *n* = 4 for IVR3, *n* = 12 for RVA3, and *n* = 10 for CRT3. ARI, activation recovery interval; AT, activation time.

Average sinus rate of animals was 102 ± 8 bpm and ventricular activation occurred rapidly (QRS interval: 66.5 ± 5.3 ms) and synchronously (ΔAT: −1.6 ± 2.6 ms; *Table 1*). Moreover, *intra*ventricular repolarization duration appeared homogenous and showed a slightly negative AT–ARI relationship (slope: −0.4; *Figure 2A*), suggestive of slightly earlier repolarization in the latest activated regions. Correspondingly, both ventricles contracted simultaneously and synchronously (ΔOnset: 2.4 ± 8.5 and ΔTTP: 8.6 ± 28.9 ms; *Table 2*).

**Table 1:** Electrical consequences of acute AV block with different (pacing) strategies of rate control

	SR	AAVB
PP	589.8 ± 82.4	515.4 ± 102.0*
RR	587.1 ± 81.7	1084.0 ± 218.3*
QRS	66.5 ± 5.3	101.9 ± 13.1*
QT	262.3 ± 15.2	339.1 ± 38.5*
QTc	293.1 ± 20.5	333.2 ± 44.5*
JTc	232.0 ± 10.6	227.5 ± 34.3
Tp-e	34.5 ± 8.8	52.8 ± 17.6*
RV_AT_	14.3 ± 2.0	36.1 ± 12.2*
LV_AT_	12.3 ± 2.0	49.6 ± 17.8*
ΔAT	−1.6 ± 2.6	11.9 ± 22.7**
RV MAPD	192.8 ± 10.4	236.7 ± 27.8*
LV ARI	213.9 ± 14.6	278.1 ± 19.8*
ΔMAPD	21.1 ± 14.1	42.6 ± 21.6***
RV STV	0.4 ± 0.2	0.9 ± 0.6**
LV STV	0.6 ± 0.3	1.0 ± 0.9

Values are represented as mean ± SD.

*N* = 51 for SR (*n* = 14 for intracardiac measurements), *n* = 51 for AAVB (*n* = 26 for intracardiac measurements).

ARI, activation recovery interval; LV_AT_, left ventricular activation time; LV STV, Short-term variability of repolarization; MAPD, monophasic action potential duration; RV, right ventricle; RV_AT_, right ventricular activation time; RV STV, short-term variability of repolarization; SR, sinus rhythm; Tp-e, time between the peak and the end of the T-wave.

*P < 0.001 vs. sinus rhythm.

**P < 0.05 vs. sinus rhythm.

***P < 0.01 vs. sinus rhythm.

**Table 2:** Mechanical effects of chronic RVA and CRT pacing

	SR	IVR	RVA	CRT
TTP (ms)				
ȃFree-wall	263.4 ± 58.1	290.5 ± 13.2	380.3 ± 105.2*	307.9 ± 39.3
ȃSeptum	272.0 ± 51.6	269.8 ± 54.5	297.6 ± 43.2	316.3 ± 39.3
ȃΔTTP	−8.6 ± 28.9	55.8 ± 20.0	82.7 ± 106.2*	−8.5 ± 31.2***
PS (%)				
ȃFree-wall	24.2 ± 7.7	47.4 ± 13.3*	22.9 ± 8.6*	34.4 ± 7.5
ȃSeptum	25.7 ± 7.2	37.4 ± 2.8	28.3 ± 8.5	36.0 ± 13.2
ȃΔPS	−1.5 ± 7.7	10.0 ± 10.8	−5.4 ± 8.5	−1.6 ± 14.1
Onset (ms)				
ȃFree-wall	10.8 ± 13.1	61.8 ± 62.1	74.2 ± 61.2	37.6 ± 60.3
ȃSeptum	8.4 ± 12.2	8.7 ± 10.2	7.3 ± 9.0	45.2 ± 52.3*
ȃΔOnset	2.4 ± 8.5	53.1 ± 62.9	66.8 ± 61.0*	−7.6 ± 37.0***

Values are represented as mean ± SD.

*N* = 13 for SR, *n* = 4 for IVR, *n* = 6 for RVA and *n* = 10 for CRT.

CRT, cardiac resynchronization therapy; IVR, idioventricular rhythm; PS, peak strain; RVA, right ventricular apex paced; SR, sinus rhythm; TTP, time-to-peak.

*P < 0.05 vs. SR.

***P < 0.05 vs. RVA3.

**Figure 3:**
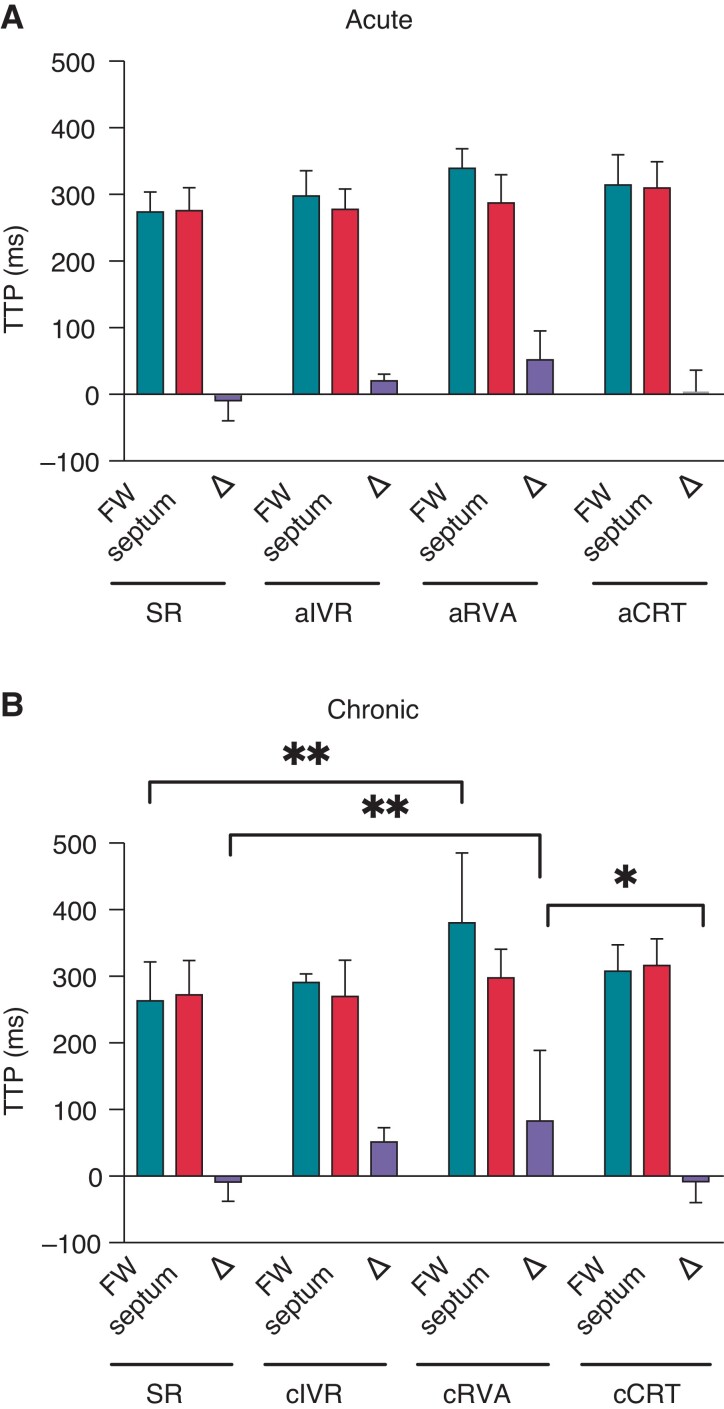
TTP of LV radial strain under different conditions. Radial strain TTP of LV free-wall (FW; yellow), septal-wall (septum; green), and the difference herein calculated as TTP FW—TTP septum (Δ; pink) following acute AV block (*A*) and after chronic exposure to bradycardic idioventricular rhythm (cIVR), RV apex (cRVA) paced or biventricular paced (cCRT) conditions (*B*). *N* = 13 for SR, *n* = 4 for IVR, *n* = 6 for RVA and *n* = 10 for CRT. FW, free-wall; SR, sinus rhythm; TTP, time-to-peak.

Creation of AV block acutely decreased heart rate, slowed conduction (QRS: 101.9 ± 13.1 ms, *P* < 0.001 vs. SR) and induced interventricular dyssynchrony in activation (ΔAT: 11.9 ± 22.7 ms, *P* < 0.05 vs. SR; *Table 1*; Supplementary material online, *Table S1*). Moreover, the AT–ARI slope became slightly positive, suggesting that repolarization became longest in the latest activated regions (*Figure 2A*). These electrical changes were mirrored by changes in timing of ventricular contraction, ΔOnset increasing from 2.4 ± 8.5 ms in SR to 76.8 ± 75.9, 32.6 ± 61.5 and −36.3 ± 81.1 ms in IVR, RVA, and CRT animals, respectively (all non-significant vs. SR; Supplementary material online, *Table S2*). Moreover, although synchrony of contraction also seemed acutely affected by AVA, this effect appeared less in CRT animals (ΔTTP from −8.6 ± 28.9 ms in SR to 20.0 ± 10.0, 51.8 ± 43.3 and 4.2 ± 31.9 ms in IVR, RVA, and CRT animals, respectively, *P* < 0.05 for RVA vs. SR; *Figure 3A*, Supplementary material online, *Table S2*).

### Chronically altered ventricular activation

**Figure 4:**
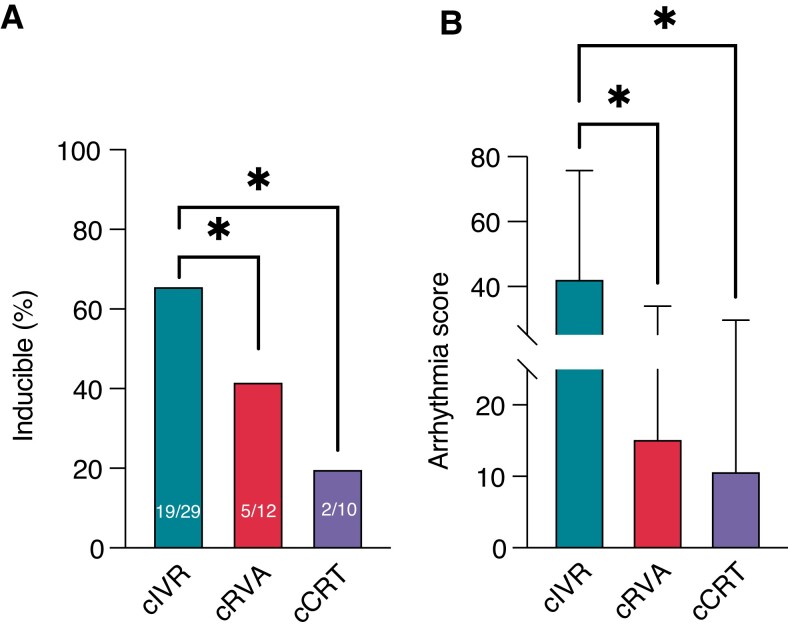
TdP inducibility andAS upon dofetilide administration following 3 weeks of remodelling. (*A*) Following 3 weeks of remodelling, 66% of IVR animals became TdP inducible, compared with 42 and 20% in the RVA and CRT-paced groups, respectively. (*B*) In comparison to RVA and CRT, arrhythmic events were more severe in IVR animals. *P < 0.05.

Chronically AVA induced different extents of pro-arrhythmic remodelling in the three groups. With just 20%, TdP inducibility was lowest in the cCRT group (2/10 animals). In cRVA animals, inducibility was more than twice as likely as cCRT animals, as 42% became TdP inducible (5/12 animals). However, cIVR caused most TdP inducibility as 66% of animals were inducible for TdP (19/29 animals; *P* < 0.05 vs. *c*RVA and cCRT; *Figure 4*; *Table 3*). Correspondingly, dofetilide caused less increase in AS in cCRT and cRVA groups (to 10.9 ± 18.8 and 15.4 ± 18.9, respectively) than in cIVR animals (to 42.8 ± 33.0, *P* < 0.05 vs. cRVA and cCRT; *Figure 4*; *Table 3*).

**Table 3:** Electrical consequences of chronic altered ventricular in IVR, RVA or CRT conditions

Baseline	IVR	RVA	CRT
ȃPP	560.3 ± 68.8	516.3 ± 77.2	533.5 ± 53.8
ȃRR	1338.0 ± 361.2	1000.0 ± 0.0*	1000.0 ± 0.0*
ȃQRS	98.8 ± 4.6	118.8 ± 10.1*	95.6 ± 5.9**
ȃQT	386.4 ± 67.0	379.6 ± 37.6	337.0 ± 24.6
ȃQTc	357.0 ± 56.5	379.6 ± 37.6	337.0 ± 24.6
ȃJTc	258.2 ± 56.2	260.8 ± 35.3	241.3 ± 24.6
ȃTp-e	82.6 ± 35.2	67.2 ± 15.0	61.9 ± 17.5
ȃRV_AT_		26.9 ± 4.6	46.0 ± 6.1*
ȃLV_AT_	35.8 ± 12.8	64.6 ± 8.3*	43.6 ± 8.3**
ȃΔAT		37.6 ± 8.1	−2.4 ± 9.6**
ȃRV MAPD		242.1 ± 16.0	221.6 ± 15.7**
ȃLV ARI	324.8 ± 51.0	271.2 ± 24.1*	266.0 ± 21.2*
ȃΔMAPD		30.0 ± 12.7	44.4 ± 14.7
ȃRV STV		1.1 ± 0.3	1.4 ± 0.7
ȃLV STV	3.2 ± 1.1	1.1 ± 0.7	0.7 ± 0.2*
Dofetilide			
ȃPP	713.8 ± 120.3***	809.7 ± 141.8***	885.9 ± 166.6*,***
ȃRR	1501.0 ± 333.2***	1000.0 ± 0.0*	1000.0 ± 0.0*
ȃQRS	100.9 ± 4.6***	119.6 ± 9.8*	96.9 ± 6.0**,***
ȃQT	579.7.3 ± 85.7***	612.0 ± 84.4***	565.6 ± 77.5***
ȃQTc	536.0 ± 81.7***	612.0 ± 84.4*,***	565.6 ± 77.5***
ȃJTc	435.2 ± 81.8***	492.5 ± 83.3***	468.8 ± 76.7***
ȃTp-e	177.2 ± 46.2***	174.2 ± 52.3***	172.8 ± 51.8***
ȃRV MAPD		359.0 ± 33.9***	328.2 ± 53.2***
ȃLV ARI	448.0 ± 48.5***	474.6 ± 59.0***	455.7 ± 42.7***
ȃΔMAPD		115.5 ± 45.5***	127.5 ± 47.7**,***
ȃRV STV		1.6 ± 0.6	1.8 ± 1.3
ȃLV STV	4.7 ± 0.9	3.9 ± 1.4***	4.2 ± 1.4***
ȃInducibility (%)	66 (19/29)	42 (5/12)*	20 (2/10)*
ȃAS	42.8 ± 33.0	15.4 ± 18.9*	10.9 ± 18.8*

Values are represented as mean ± SD.

*N* = 29 for IVR (*n* = 4 for intracardiac measurements), *n* = 12 for RVA and *n* = 10 for CRT.

ARI, activation recovery interval; AS, arrhythmia score; CRT, cardiac resynchronization therapy; IVR, idioventricular rhythm; LVAT, left ventricular activation time; MAPD, monophasic action potential duration; LV STV, Short-term variability of repolarization; RV, right ventricle; RVA, right ventricular apex paced; RVAT, right ventricular activation time; RV STV, short-term variability of repolarization; ΔAT, interventricular differences in activation time (calculated as LV_AT_ – RV_AT_); ΔMAPD, interventricular dispersion of repolarization (calculated as LV ARI–RV MAPD).

*P < 0.05 vs. IVR.

**P < 0.05 vs. RVA.

***P < 0.05 vs. baseline.

Mechanically, each group exhibited different extents of MD (*Table 2*). Most notably, this interventricular dyssynchrony in timing and force of contraction was lowest in cCRT animals (ΔTTP: −8.5 ± 31.2 ms in cCRT vs. 55.8 ± 20.0 ms in cIVR and 82.7 ± 106.2 ms in the cRVA animals; *P* < 0.05 RVA vs. CRT; *Figure 3B*; *Table 2*). Moreover, although contraction was delayed in cCRT animals, the synchrony and force of contraction appeared very similar to SR conditions (ΔTTP: −8.6 ± 28.9 ms in SR; ΔPS: −1.5 ± 7.7% and −1.6 ± 14.1% in SR and cCRT animals, respectively, all *non-significant; Table 2*).

Electrically, ventricular activation was significantly more synchronous in cCRT than in cRVA animals (ΔAT: −2.4 ± 9.6 vs. 37.6 ± 8.1 ms, respectively, *P* < 0.05). Moreover, all groups showed a negative AT-RT slope (as in SR), which became increasingly steeper from cCRT to cRVA to cIVR animals (*Figure 2*). Dofetilide further unmasked this *intra*ventricular heterogeneity in repolarization reserve, as regardless of rate strategy, repolarization prolonged most in the earliest activated regions. This steepening of the slope was least pronounced in the least-inducible group of cCRT animals (slope: −0.7) in comparison to cRVA and cIVR animals (slope: −4.5 and −3.2, respectively). Additionally, administration of dofetilide prolonged PP-intervals in all animals, but the extent of this prolongation also differed between each group; the prolongation becoming increasingly more pronounced going from cIVR to cRVA to cCRT animals (PP-interval following dofetilide to 713.8 ± 120.3, 809.7 ± 141.8 and 885.9 ± 166.6 ms in cIVR, cRVA, and cCRT animals, respectively; *P* < 0.05 for cIVR vs. cCRT; *Table 3*).

## Discussion

In this study, we demonstrate that development of TdP susceptibility in the CAVB dog model is correlated with extent of electromechanical dyssynchrony, which is induced by AVA. We showed that TdP susceptibility was lowest in cCRT animals, which were characterized by (i) less mechanical ventricular dyssynchrony and (ii) less LV intraventricular dispersion of repolarization.

### Arrhythmogenic vulnerability caused by mechanical discoordination

Multiple studies have suggested that chronic RVA pacing progresses LV dysfunction as a result of electrical, and consequentially, MD.^15^ Correspondingly, biventricular pacing, which enhances synchrony of ventricular activation, is superior to RVA pacing in preserving LV function.^7^ As electrical remodelling is initiated to improve contractile function, the extent wherein this occurs seems to correlate with the severity of MD. Electrical remodelling under chronic RVA conditions is therefore more extensive than under chronic CRT conditions, resulting in more dispersion of repolarization and increased arrhythmic susceptibility. Also in CAVB dogs, 3 weeks of remodelling partially reverses RVA pacing-induced MD, although TdP susceptibility increases.^7^ Hence, MD appears to induce ventricular (electrical) remodelling, which improves synchrony of contraction, but adversely increases TdP susceptibility. Similarly, suboptimal CRT implantation could initiate MD and thereby induce pro-arrhythmic remodelling in CRT non-responders.^4,5^

In this study, we exposed CAVB dogs to chronically unpaced (cIVR), cRVA or cCRT conditions, to compare arrhythmic outcomes of remodelling under different conditions of AVA and thus extents of MD. Notably, in IVR animals, focus of activation is uncontrolled, unstable and originates from three predilected sites.^16^ Although activation is controlled in RVA and CRT animals, this unnatural activation pattern naturally results in MD, whereas its inconsistency is difficult to adapt to. Following chronically altered activation, several electrical parameters, including LV STV, were significantly different between the groups. However, these differences, reflective of different degrees of remodelling, became even clearer upon administration of dofetilide, and made most evident by TdP susceptibility of each group. Although TdP susceptibility was highest in IVR, CRT animals were significantly less vulnerable to TdP. Cardiac resynchronization therapy animals also displayed the most synchronous LV contraction on echocardiography, closely resembling SR conditions. Hence, although all groups were exposed to AVA, the extent of continuous and chronic MD that persists may determine the extent of ventricular pro-arrhythmic electromechanical remodelling. However, whether the electrical or mechanical changes are the primary drive of remodelling remains unclear.

### Ventricular electrical remodelling

In the CAVB dog model, electrical remodelling induces changes in Ca^2+^-handling and downregulation of potassium channels.^8^ Consequently, administration of *I_Kr_*-blocker dofetilide further impedes cardiac electrical stability by reducing repolarization reserve, which is quantified by STV.^8,14^ This study showed that ventricular remodelling, initiated by chronically altered activation and bradycardia, reduces repolarization reserve and thereby increases TdP susceptibility (*Table 3*).

This study also assessed electrical remodelling in the *spatial* dimension, using the AT–ARI slope.^7^ Similar to previous studies, dofetilide steepened the negative AT–ARI slope, suggesting that repolarization reserve reduces most in early activated regions as they show relatively more dofetilide-induced ARI prolongation.^7^ This steeper slope reflects more *intra*ventricular dispersion of repolarization, which is also inverted compared with acute AV block, and has been implicated in both the development and perpetuation of longer-lasting TdP.^7,17,18^ Additionally, this study showed that regardless of ventricular activation pattern, dofetilide causes the largest repolarization prolongation in early activated regions, indicating that electrical remodelling is driven by differences in AT and/or the consequential MD.

### Neural remodelling

Previous studies have demonstrated that sympathetic modulation promotes arrhythmogenesis in the CAVB dog model.^19^ In this study, dofetilide-induced increases in PP-interval were significantly less in IVR animals compared with RVA and CRT animals. This suggests that AV block induced neural responses could be proportional to the degree of MD; a stronger neural response (as possibly present in IVR animals), resulting in more sympathetic activation and therefore more pro-arrhythmic conditions. However, this correlation remains hypothetical and the relationship between mechanical dysfunction and neural remodelling warrants further investigation.

### Clinical relevance

This study showed that extent of electromechanical dyssynchrony relates to the development of arrhythmogenic vulnerability. It therefore highlights the importance of avoiding dyssynchrony in structurally remodelled hearts and potentially explains results of the Echocardiography Guided Cardiac Resynchronization Therapy (EchoCRT) study. This study, which was prematurely terminated due to futility,^20^ reported a tendency towards increased mortality in HF patients without a prolonged QRS (≤130 ms) who underwent CRT therapy. Potentially, CRT induced electromechanical dyssynchrony, which resulted in pro-arrhythmic cardiac remodelling.^20^ Hence, though CRT reduces morbidity and mortality in carefully selected HF patients,^2^ inappropriate CRT implantation can be detrimental. Importantly, these findings are also relevant in the setting of left bundle-branch block pacing and His-bundle pacing.

### Limitations

Due to arrhythmic activity, electrophysiological data following infusion of dofetilide in cIVR, cRVA and cCRT experiments are taken at different timepoints. As less dofetilide was administered at earlier timepoints, this could have caused underestimation of the changes herein.

In IVR animals, dofetilide was administered under more bradycardic conditions. Bradycardia promotes arrhythmogenesis in the CAVB dog model^10^ and might have contributed to the increased TdP susceptibility.

Echocardiography and electrophysiological studies with an LV EGM were performed in only four IVR animals. Although limited, these results adequately represent mechanical and electrical dyssynchrony in these animals. Especially since in IVR, the focus is inconsistent and arising from three different predilected sites, reliable quantification of dyssynchrony is challenging.

## Conclusion

In the CAVB dog model, AVA contributes to pro-arrhythmic remodelling as the degree of electromechanical dyssynchrony correlates with the arrhythmogenic outcome.

## Supplementary Material

euac164_Supplementary_Data

## Data Availability

The data underlying this article will be shared on reasonable request to the corresponding author.
