# Supplementary material for: Chronically altered ventricular activation causes pro-arrhythmic cardiac electrical remodelling in the chronic AV block dog model
Source: Europace. 2022 Sep 20;25(2):707–15. doi: 10.1093/europace/euac164 (PMC9934998; doi:10.1093/europace/euac164)
Supplement: euac164_Supplementary_Data [file euac164_supplementary_data.zip › Supplementary table 1.docx]

**Supplementary table 1: Electrical consequences of acute AV-block with different (pacing) strategies of rate control**

| *Baseline* | | | *SR* | *aIVR* | | | *aRVA* | *aCRT* | |
| --- | --- | --- | --- | --- | --- | --- | --- | --- | --- |
| PP | | | 589.8 ± 82.4 | 514.6 ± 112.9* | | | 509.0 ± 81.1* | 525.4 ± 101.2 | |
| RR | | | 587.1 ± 81.7 | 1149.0 ± 276.3* | | | 1000.0 ± 0.0* | 1000.0 ± 0.0* | |
| QRS | | | 66.5 ± 5.3 | 95.8 ± 6.9* | | | 121.1 ± 8.1*† | 95.8 ± 7.3*^‡^ | |
| QT | | | 262.3 ± 15.2 | 315.3 ± 30.2* | | | 378.3 ± 25.1*† | 358.6 ± 18.1*†^‡^ | |
| QTc | | | 293.1 ± 20.5 | 302.3 ± 27.5 | | | 378.3 ± 25.1* | 358.6 ± 18.1* | |
| JTc | | | 232.0 ± 10.6 | 206.5 ± 25.8* | | | 257.2 ± 25.8*† | 263.0 ± 16.0† | |
| Tp-e | | | 34.5 ± 8.8 | 49.8 ± 21.6* | | | 58.9 ± 10.8*† | 54.2 ± 2.6* | |
| RV AT | | 14.3 ± 2.0 | | | 40.0 ± 30.7* | 27.4 ± 5.3* | | | 47.1 ± 6.5*^‡^ |
| LV AT | Mean | 12.3 ± 2.0 | | | 28.9 ± 29.0 | 61.5 ± 9.6*† | | | 43.6 ± 8.2*^‡^ |
|  | Apex | 8.7 ± 4.3 | | | 27.2 ± 30.7* | 56.4 ± 10.2*† | | | 42.1 ± 8.8*†^‡^ |
|  | Free wall | 12.3 ± 3.4 | | | 27.7 ± 29.9* | 67.0 ± 13.1*† | | | 35.4 ± 6.8*^‡^ |
|  | Septum | 16.3 ± 11.1 | | | 31.3 ± 20.4 | 57.5 ± 12.8*† | | | 56.5 ± 16.2*† |
| **Δ**AT | | -1.6 ± 2.6 | | | -11.1 ± 13.1 | 35.6 ± 8.7*† | | | -3.2 ± 5.7^‡^ |
| RV MAPD | | 192.8 ± 10.4 | | | 252.5 ± 46.2* | 240.2 ± 27.8* | | | 228.9 ± 21.7* |
| LV ARI | Mean | 213.9 ± 14.6 | | | 296.7 ± 27.6* | 276.7 ± 18.3* | | | 272.3 ± 15.2* |
|  | Apex | 219.5 ± 20.0 | | | 289.3 ± 17.9* | 273.8 ± 22.2* | | | 283.1 ± 16.0* |
|  | Free wall | 210.5 ± 15.5 | | | 292.3 ± 25.4* | 281.5 ± 12.5* | | | 276.1 ± 16.1* |
|  | Septum | 215.4 ± 16.2 | | | 299.3 ± 29.9* | 285.7 ± 20.1* | | | 268.6 ± 27.5* |
| **Δ**MAPD | | 21.1 ± 14.1 | | | 44.1 ± 33.9* | 36.4 ± 16.7 | | | 43.5 ± 22.1* |
| RV STV | | 0.4 ± 0.2 | | | 0.9 ± 0.3 | 0.8 ± 0.4 | | | 0.8 ± 0.8 |
| LV STV | | 0.6 ± 0.3 | | | 0.8 ± 0.4 | 1.3 ± 1.2 | | | 0.6 ± 0.2 |

Values are represented as mean ± SD.

* *p*<0.05 *vs.* Sinus Rhythm

† *p*<0.05 *vs.* IVR

^‡^ *p*<0.05 *vs.* RVA

SR: Sinus rhythm

IVR: Idioventricular rhythm

RVA: Right ventricular apex paced

CRT: Biventricular paced

RV AT: Right ventricular activation time

LV AT: Left ventricular activation time

ΔAT: Interventricular differences in activation time (LV AT – RV AT)

LV ARI: Left ventricular activation recovery interval

RV MAPD: Right ventricular monophasic action potential duration

ΔMAPD: Interventricular dispersion of repolarization (LV ARI – RV MAPD)

RV STV: Right ventricular short-term variability of repolarization

LV STV: Left ventricular short-term variability of repolarization
