# Supplementary material for: Chronically altered ventricular activation causes pro-arrhythmic cardiac electrical remodelling in the chronic AV block dog model
Source: Europace. 2022 Sep 20;25(2):707–15. doi: 10.1093/europace/euac164 (PMC9934998; doi:10.1093/europace/euac164)
Supplement: euac164_Supplementary_Data [file euac164_supplementary_data.zip › Supplementary table 2.docx]

**Supplementary table 2: Mechanical consequences of acute AV-block with different (pacing) strategies of rate control**

|  |  | *SR* | *aIVR* | *aRVA* | *aCRT* |
| --- | --- | --- | --- | --- | --- |
| *TTP (ms)* | Free wall | 263.4 ± 58.1 | 297.7 ± 37.9 | 339.1 ± 29.5* | 314.1 ± 45.9 |
|  | Septum | 272.0 ± 51.6 | 277.7 ± 30.6 | 287.3 ± 42.5 | 309.9 ± 39.1 |
|  | ΔTTP | -8.6 ± 28.9 | 20.0 ± 10.0 | 51.8 ± 43.3* | 4.2 ± 31.9^‡^ |
| *PS (%)* | Free wall | 24.2 ± 7.7 | 36.9 ± 9.4 | 28.9 ± 5.1 | 24.8 ± 7.0 |
|  | Septum | 25.7 ± 7.2 | 24.6 ± 10.9 | 25.9 ± 6.6 | 24.5 ± 7.0 |
|  | ΔPS | -1.5 ± 7.7 | 12.3 ± 6.5 | 3.0 ± 9.4 | 0.3 ± 10.8 |
| *Onset (ms)* | Free wall | 10.8 ± 13.1 | 103.5 ± 66.8 | 60.8 ± 59.5* | 47.4 ± 49.4 |
|  | Septum | 8.4 ± 12.2 | 26.7 ± 15.3 | 28.2 ± 13.0 | 83.7 ± 71.2* |
|  | ΔOnset | 2.4 ± 8.5 | 76.8 ± 75.9 | 32.6 ± 61.5 | -36.3 ± 81.1† |

Values are represented as mean ± SD.

* *p*<0.05 *vs.* Sinus Rhythm

† *p*<0.05 *vs.* IVR

^‡^ *p*<0.05 *vs.* RVA

SR: Sinus rhythm

RVA: Right ventricular apex paced

CRT: Biventricular paced

TTP: Time to peak

PS: Peak strain

Onset: Onset of radial strain

ΔTTP: Intraventricular difference in time to peak (TTP_free wall_ – TTP_septum_)

ΔPS: Intraventricular difference in peak strain (PS_free wall_ – PS_septum_)

ΔOnset: Intraventricular difference in onset of radial contraction (Onset_free wall_ – Onset_septum_)
